# Dual Role of Diallyl Disulfide (DADS) on Invasive Potential and β-Catenin Dynamics in HER2-Positive Breast Cancer Cells

**DOI:** 10.3390/cancers17213572

**Published:** 2025-11-05

**Authors:** Marcello Dell’Aira, Silvia Grassilli, Marina Pierantoni, Valeria Bertagnolo, Federica Brugnoli

**Affiliations:** 1 Department of Translational Medicine, University of Ferrara, 44121 Ferrara, Italy; marcello.dellaira@unife.it (M.D.); marina.pierantoni@unife.it (M.P.); federica.brugnoli@unife.it (F.B.); 2 Department of Environmental Sciences and Prevention, University of Ferrara, 44121 Ferrara, Italy; silvia.grassilli@unife.it; 3 LTTA Centre, University of Ferrara, 44121 Ferrara, Italy

**Keywords:** HER2-positive breast tumor, garlic derivatives, Akt signaling, β-catenin dynamic

## Abstract

**Background/Objectives:** Natural compounds are being increasingly explored as potential adjuvants to conventional drugs in oncological treatments. Regarding breast tumors, several studies indicate that garlic (*Allium sativum*) may protect against onset, counteracts aggressiveness, and prevents malignant progression of cells from non-invasive tumors. It has been widely demonstrated that garlic derivatives induce apoptosis and reduce invasive potential in ER-positive and triple-negative breast tumor cells. However, the current literature lacks studies investigating their effects on HER2-positive (HER2+) breast cancers. This study therefore aimed to explore the effects of a garlic extract and diallyl disulfide (DADS), one of its most bioactive organosulfur compounds, on HER2+ phenotype breast tumor cells. **Methods:** The effects of a garlic extract and diallyl disulfide (DADS) were investigated on MDA-MB-453 and SKBR3 breast tumor cell lines. Cell growth, invasive potential, and Akt-related signaling were assessed after 4–72 h of garlic derivatives administration. The intracellular localization of β-catenin was examined with immunofluorescent confocal microscopy. **Results:** A dual role of DADS, dependent on the duration of treatment, was revealed. Acute administration induced a significant decrease in invasive potential, while prolonged treatment promoted HER2+ cell invasiveness. These effects were directly correlated with the activation of Akt and the nuclear accumulation of β-catenin, known to induce expression of genes associated with tumor malignancy. **Conclusions:** Although further investigations are needed to establish the exact mechanism and to assess the in vivo reproducibility of these phenomena, our results highlight the heterogeneous response to natural compounds of complex diseases like cancer.

## Simple Summary

Natural compounds are increasingly studied for their potential anti-cancer properties. While garlic derivatives show promise against some breast cancer phenotypes, their effects on HER2-overexpressing breast tumors remain unknown. This study investigated how diallyl disulfide (DADS), one of most bioactive garlic compounds, and a DADS-enriched garlic extract, influence the invasive behavior of HER2-expressing breast cancer cells. We revealed a dual response of HER2+ cells to DADS; short-term exposure reduced cell invasiveness, while prolonged treatment, unexpectedly, increased their invasive potential. The DADS-induced modulation of the Akt/β-catenin signaling seems to be at the basis of these events. Our results highlight that natural compound interactions with cancer cells can be highly heterogeneous and underscore the need for careful research into an often-overlooked aspect, that is the safety of using natural compounds in the context of complex diseases such as cancer.

## 1. Introduction

Breast cancer (BC) is a heterogeneous disease constituting about one-third of cancers in women, with unique epidemiological patterns, a mortality rate of 15% of diagnosed cases, and an onset influenced by genetic, environmental, and lifestyle factors [1]. Breast tumors exhibit variable biological and molecular characteristics, affecting their clinical behavior and treatment responses. Tumor classification is at the basis of accurate diagnosis and oncological decision-making, and primary evaluation includes the expression of Estrogen Receptor (ER), Progesterone Receptor (PR), and Human Epidermal Growth Factor Receptor 2 (HER2), which are indicative markers for hormonal and/or anti-HER2 therapies [2]. The advent of technologies that explore tumors at genomic levels allowed researchers to identify, inside the principal BC phenotypes, molecular subtypes with peculiar responses to treatments. The BC that has improved its prognosis the most in the last few years is the one overexpressing HER2, which constitutes 20% of invasive mammary tumors and is associated with an aggressive clinical course, even if responds to anti-HER2 treatments [3]. Trastuzumab was the first humanized antibody against HER2 which achieved remarkable success, and current approaches still involve chemotherapy combined with anti-HER2 antibodies. More recently, further HER2-targeted therapies have been developed, such as bispecific antibodies, drug-conjugated antibodies (ADCs), and Tyrosine Kinase Inhibitors (TKIs) [4,5] which, despite their anti-tumor efficacy, can give rise to toxicity and impact patients’ quality of life. Unluckily, about 20% of patients with localized disease still experience resistance to HER2-targeted therapies [4], due to compromised binding of antibodies to HER2, mutations in HER2/ERBB2, or therapy-induced increase in HER2 expression [6,7,8,9,10,11].

An important factor influencing resistance mechanisms in breast tumors is their heterogeneity. It was reported that 10% of HER2+ tumors showed variable levels of the receptor, significantly impacting the response to HER2-targeted therapies [12]. It was shown that the complete pathological response after neoadjuvant treatment with Trastuzumab emtansine (T-DM1) was 55% in patients with HER2 homogeneous tumors, compared to 0% in those with heterogeneous tumors [13]. Another significant factor in resistance to neoadjuvant HER2-targeted therapies is the presence of PIK3CA mutations, which are found in 20% of HER2+ tumors [14,15]. Accordingly, CLEOPATRA and EMILIA clinical studies indicate that PIK3CA mutations are a poor prognostic marker in HER2+ metastatic breast cancer [16,17].

Explored approaches to overcoming drug resistance in patients treated with HER2+ tumors involve the Wnt/β-catenin signaling pathway, which regulates epithelial-to-mesenchymal transition (EMT), promoting tumor invasion and metastasis, and contributing to drug resistance [18,19]. The Wnt signaling pathway is highly activated in trastuzumab-resistant breast cancer cells, suggesting the inhibition of the Wnt/β-catenin pathway as a promising therapeutic strategy to improve the effectiveness of HER2-targeted treatments [19,20].

Due to their favorable safety, and their onco-suppressor roles in breast cancer and other malignancies, emerging strategies to improve the life quality of breast cancer patients include the use of bioactive natural compounds [21,22]. Phytochemicals with anti-tumor activity have been identified in many edible plants, including soy, tomato, broccoli, and *Allium* vegetables like garlic [23]. High garlic intake is associated with a protective effect against various tumors, including breast cancer [24], and garlic derivatives, mainly organosulfur compounds, have become attractive as anti-cancer agents due to their ability to induce apoptosis in vitro [25] and to inhibit tumor formation and growth in vivo [26,27,28]. In breast cancer cells with an ER+ and triple-negative (TNBC) phenotype, garlic compounds act on cyclins, and/or on MAPK, ERK, AKT, and EMT pathways [29,30]. Recent studies also demonstrated that garlic has a protective role against the hypoxia-induced malignant progression of non-invasive breast cancer cells [31], and exerts a selective down-modulation of invasive potential in cells from Patient Derived Xenograft (PDX) with TNBC, reflecting the intra-phenotypic heterogeneity of this breast tumor phenotype [32].

Despite promising results on the use of natural compounds in breast tumors, there are no studies exploring the impact of garlic on HER2+ cancers. We reported, here, the first data on the effects of garlic, particularly of diallyl disulfide (DADS), one of its most bioactive compounds, on breast tumor cells expressing HER2, to fill the knowledge gap and to establish the potential usefulness of garlic derivatives in a breast cancer phenotype which, despite the significant progresses made in recent years, presents some important critical issues.

## 2. Materials and Methods

All reagents were obtained from Merck KGaA (Darmstadt, Germany) unless otherwise indicated.

### 2.1. Cells and Treatments

The breast cancer-derived MDA-MB-453 and SKBR3 cell lines were acquired from the American Type Culture Collection (Rockville, MD, USA) and were cultured in Dulbecco’s modified Eagle’s medium (DMEM, Gibco Laboratories, Grand Island, NY, USA) supplemented with 10% fetal bovine serum (FBS, Gibco Laboratories) and 1% penicillin–streptomycin solution (Gibco Laboratories). All cell lines were maintained at 37 °C in a humidified atmosphere and evaluated monthly for mycoplasma and other contaminations.

A hydro-alcoholic garlic extract was prepared following a previously described procedure [31,32], subjected to lyophilization, and resuspended in PBS (0.25 g/mL) before treatments, as previously described [33].

Both cell lines were treated for 72 h with 1:800 (*v*/*v*) of 0.25 g/mL lyophilized garlic extract (LYO), and subjected to evaluation of cell cycle, apoptosis, and invasive potential.

SKBR3 cells were also treated for 4, 24, 48 and 72 h with DADS (SMB00378) solubilized in DMSO, at final concentrations of 200 µM, 400 µM and 800 µM.

The allosteric Akt inhibitor MK-2206 (S1078, Selleck Chemicals, Houston, TX, USA), dissolved in DMSO, was administered to SKBR3 cells for 72 h at final concentrations of 6 µM and 12 µM.

In all experimental conditions, cell viability was evaluated with the Trypan Blue Exclusion Test and inverted phase-contrast microscope (Diaphot, Nikon, Melville, NY, USA) analysis.

### 2.2. Apoptosis and Cell Cycle Analysis

Apoptosis was evaluated using the Annexin A5-FITC Kit (IM3546, Beckman Coulter Life Sciences, Milan, Italy), according to the instructions of the manufacturer. Briefly, 2.5 × 10^5^ cells were washed with PBS and incubated with 50 μL of MIX Solution, containing Binding 1X, Annexin V, and propidium iodide (PI), for 15 min in the dark at 4 °C. Each sample was then added with 90 μL of Binding 1X and analyzed with the FACS Calibur flow cytometer (BD Biosciences, San Jose, CA, USA).

For analysis of cell cycle distribution, 2.5 × 10^5^ cells were washed in PBS, fixed with 70% ethanol, and incubated in the dark at room temperature for 30 min with 100 μg/mL RNAse and 20 μg/mL PI, following the previously reported procedure [33]. The fluorescence of individual nuclei was detected using a FACS Calibur flow cytometer (BD Biosciences). The ratio of cells in the G0/G1, S and G2/M phases was calculated by the CellQuest Pro 6.0 software (BD Biosciences), as previously described [33].

### 2.3. Real-Time Cell Invasion Assays

Cell invasion was evaluated with a xCELLigence Real-Time Cell Analyzer System (RTCA System, Roche Applied Science, Mannheim, Germany), developed to continuously monitor cell events in real time by measuring cell’s electrical impedance, directly inside the incubator. Briefly, 40,000 cells/well, resuspended in DMEM medium without FBS, were seeded onto the top chambers of CIM-16 plates (Roche Applied Science), electronically integrated Boyden Chambers with 8 mm pores and micro-electrodes located on the underside of the upper chamber membrane. The upper chambers were covered with a layer of 1:40 diluted Matrigel (BD Biosciences), while the bottom chambers were filled with a medium containing 10% FBS (Gibco Laboratories), exerting a chemoattractant activity.

Impedance signals were expressed as a dimensionless parameter (cell index, CI) which values were considered positive when >0.1. The evaluation of each experimental condition in every experiment was performed in triplicate, and the detection of cell index was programmed every 15 min for a total duration of 24 h, as previously described [33]. The kinetics of cell invasion were also quantified by calculating the slope: a value indicating the steepness, inclination, gradient, and changing rate of the CI curves over time.

### 2.4. Immunochemical Analysis

Total lysates from cells under the different experimental conditions were separated on 8.5% polyacrylamide denaturing gels and blotted onto nitrocellulose membranes (GE Healthcare Life Science, Little Chalfont, UK), which were reacted with antibodies directed against p-β-Catenin (sc-57535) and β-Catenin (sc-7963) from Santa Cruz Biotechnology (Heidelberg, Germany); Akt (AF2055), p-Akt (#3787S), Akt2 (#3063), p-Akt2 (Ser474, #8599), p-GSK-3β (Ser9, #5558) and GSK-3β (#9315) from Cell Signaling Technology (Danvers, MA, USA); against Akt1 (#610860, BD Biosciences), p-Akt1 (Ser473, #05-736), and β-Actin (#A4700, Merck KgaA), following the previously reported procedures [32]. The immunocomplexes were detected by using a WESTAR NOVA 2.0 kit (Cyanagen, Bologna, Italy), and the chemiluminescence-derived bands were captured with an iBright FL1500 Imaging System (Thermo Fisher Scientific, Waltham, MA, USA) and quantified with Invitrogen iBright Analysis Software, Desktop Version 5.4.0 (Thermo Fisher Scientific).

### 2.5. Immunocytochemical and Confocal Analysis

For immunofluorescence analysis, SKBR3 cells, directly grown on glass slides, were fixed with freshly prepared 4% paraformaldehyde for 10 min at room temperature. To analyze F-actin, fixed cells were washed with PBS, permeabilized with 1% Triton X-100 in PBS for 5 min at room temperature, incubated with PBS added with 1% BSA for 30 min, and then stained with tetramethyl rhodamine isothiocyanate (TRITC)-conjugated phalloidin in PBS for 30 min at room temperature in the dark. Subsequently, all samples were incubated with 0.5 µg/mL 4′,6-diamidino-2-phenylindole (DAPI), dried with ethanol, and mounted in glycerol containing 1,4-diazabicyclo [2.2.2] octane (DABCO) to delay fading. Samples were analyzed with a Nikon Ci-L microscope (Nikon) equipped with a DS-Qi2Mc digital camera (Nikon). Fluorescent images were captured by NIS-Elements BR Imaging Software 5.20.20 version (Nikon), regions containing approximately 50–100 cells were selected and the fluorescence intensity per cell was quantified, excluding non-specific signals, as previously reported [34].

To evaluate β-catenin intracellular distribution, fixed cells were reacted with the antibody against β-catenin for 3 h at room temperature in Net Gel solution (150 mM NaCl, 5 mM EDTA, 50 mM Tris-HCl pH 7.4, 0.05% NP40, 0.25% Carrageenan Lambda gelatin, and 0.02% Na azide), and then labeled with a fluorescein isothiocyanate (FITC)-conjugated secondary antibody (Thermo Fisher Scientific) in the dark at room temperature, as previously reported [33]. After three washes with PBS, cells on coverslips were dried with ethanol, mounted in glycerol-DABCO, and analyzed with an Olympus FV3000 confocal microscope (Olympus Europe, Hamburg, Germany), which was equipped with a 63× oil immersion objective (N.A. 1.4), by taking z-series of 0.42 μm each to capture the entire volume of the cells. To evaluate β-catenin nuclear staining, confocal digitized images were analyzed with ImageJ software 1.54 g version (http://rsb.info.nih.gov/ij/, accessed on 28 February 2025).

### 2.6. Survival Outcomes and β-Catenin Levels

TRGAted (https://nborcherding.shinyapps.io/TRGAted/, accessed on 13 March 2025) was used to explore the relationships between β-catenin levels and progression-free survival (PFS) in HER2+ breast cancers from The Cancer Genome Atlas (TCGA) [35]. Kaplan–Meier curves were generated by selecting the type of cancer (breast), the subtype (HER2+), the protein of interest (β-catenin), the survival type curves (PFS), the tumor stages (all), the histological types (all), and the age groups (all). Survival curves were generated by separating the protein levels into two groups based on the lowest *p*-value, according to Borcherding et al. [35]. The hazard ratios (HRs) were provided.

### 2.7. Statistical Analysis

Statistical analysis was performed by using a 2-tailed Student’s *t*-test for unpaired data using the GraphPad Prism 6.0 statistical package (GraphPad Software, San Diego, CA, USA). The results are expressed as means ± standard deviations of three independent experiments. *p*-values < 0.05 are considered statistically significant.

## 3. Results

### 3.1. Garlic Extract Reduces Cell Growth and Induces Invasive Potential in HER2-Overexpressing Breast Tumor Cells

In this study we utilized the MDA-MB-453 and SKBR3 breast tumor cell lines that were expressing HER2 at different levels and representing two distinct scores of the receptor profile. While MDA-MB-453 cells exhibit low HER2 [36], high expression of the receptor characterizes the SKBR3 cell line [37]. Despite their described different genomic and molecular profiles, the two cell lines show similar levels of Akt, which is strongly activated only in SKBR3 cells, reflecting their HER2 status (Supplementary Figure S1).

**Figure 1:**
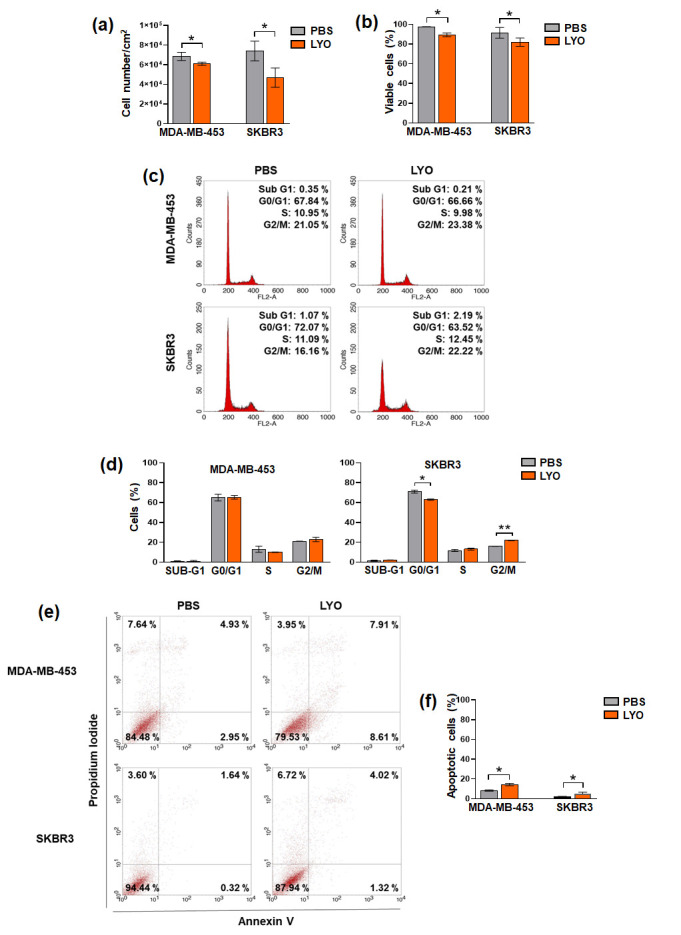
Effects of garlic extract on cell growth and apoptosis of HER2+ breast cancer cells. After 72 h of culture under control conditions (PBS) or in the presence of lyophilized garlic extract (LYO), MDA-MB-453 and SKBR3 cells were subjected to the evaluation of proliferation (**a**), viability (**b**), and to cytofluorimetric analysis of cell cycle distribution (**c**,**d**) and total (early and late) apoptosis (**e**,**f**). Results represent the mean of the three separate experiments ± SD. * *p* < 0.05; ** *p* < 0.01 compared to the control.

To evaluate the effects of the lyophilized garlic extract (LYO), both cell lines were exposed for 72 h to LYO at a concentration proven to be efficient in reducing cell growth, without high cytotoxic effects, in other breast tumor subtypes [33]. The evaluation of cell proliferation and viability indicated that LYO reduced the growth of both cell lines, with SKBR3 cells exhibiting the highest sensitivity (Figure 1a,b).

To better explore the effects of LYO on the cell growth of our cell models, we performed a cytofluorimetric analysis of cell cycle distribution, showing that only SKBR3 exhibited significant changes, with a decrease in number of cells in the G0/G1 phases, and their accumulation in the G2/M phases (Figure 1c,d). We further performed a flow cytometry evaluation of apoptosis, revealing that both treated cell lines exhibited a small but significant increase in annexin-positivity (Figure 1e,f). These data indicate that, at the used concentration, our garlic extract induced a modest decrease in cell growth without significant cytotoxic effects in HER2+ cells, as we have already demonstrated in breast tumor cells with ER+ and TNBC phenotypes [31,32,38].

**Figure 2:**
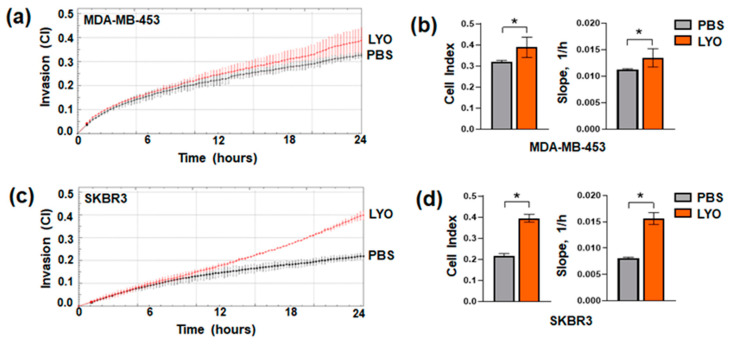
Effects of LYO on invasive potential of HER2+ breast cancer cells. Representative impedance-based xCELLigence-driven dynamic monitoring of invasion of MDA-MB-453 (**a**) and SKBR3 (**c**) cells after 72 h in control conditions (PBS) or in the presence of LYO. Curves represent mean ± SD of cell index (CI). Analysis of CI values at 24 h, and of the slope, describing the steepness, incline, gradient, and changing rate of the CI curves over time, are reported (**b**,**d**). Data represents the mean of the three separate experiments ± SD. * *p* < 0.05 compared to the control.

Tumor metastases are a significant concern and one of the leading causes of morbidity for patients with HER2+ cancers [39,40]. To investigate whether garlic extract modulates invasive capability of HER2+ breast tumor cells, both cell lines were treated with LYO for 72 h and subjected to real-time analysis with xCELLigence RTCA technology. As reported in Figure 2, the assay revealed that the two cell lines intrinsically possess low invasive capability in vitro. After treatment with garlic extract, unlike what we observed in other breast tumor phenotypes, both cell lines showed an unexpected increase in their invasive capability, with SKBR3 (Figure 2c,d) more responsive than MDA-MB-453 cells (Figure 2a,b).

### 3.2. Diallyl Disulfide Reduces Cell Growth and Induces Invasiveness in SKBR3 Breast Cancer Cells

**Figure 3:**
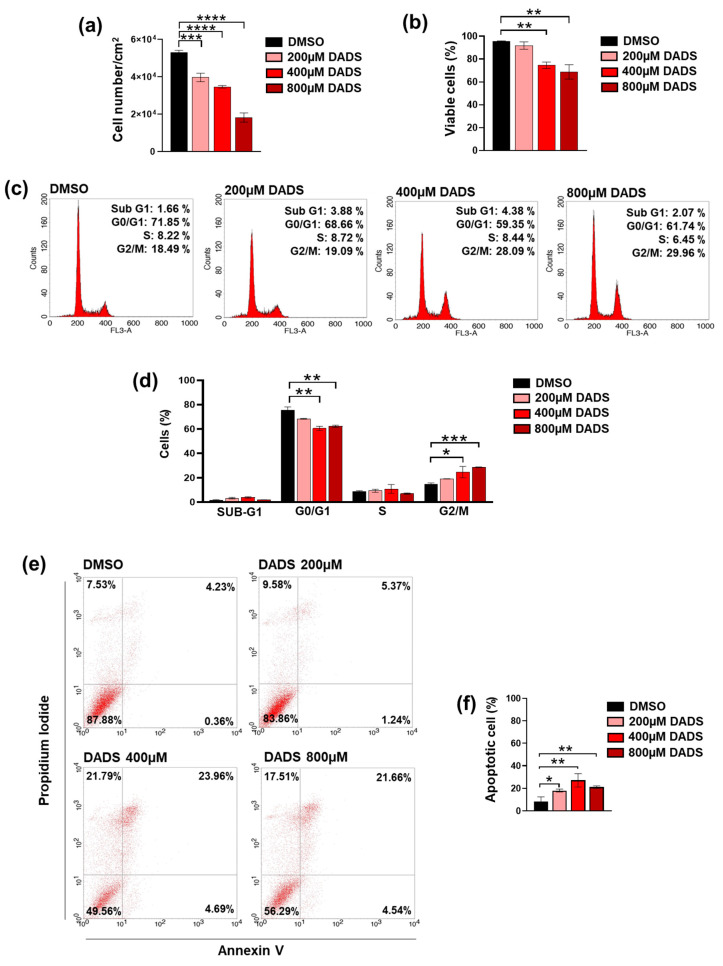
Effects of DADS on cell growth and apoptosis in SKBR3 cells. After treatment for 72 h with DADS at the indicated concentrations, cells were subjected to evaluation of proliferation (**a**), viability (**b**), cell cycle distribution (**c**,**d**), and apoptosis (**e**,**f**). Data represents the mean of the three separate experiments ± SD. * *p* < 0.05; ** *p* < 0.01; *** *p* < 0.001; **** *p* < 0.0001 compared to the control (DMSO).

Based on the above reported data, we further explored the effects of garlic derivatives on SKBR3 cells, which is the one of the two cell lines in which our extract showed the greatest effects in up-modulating invasive potential. To assess whether the effects of LYO were indeed due to its best-known components, we treated SKBR3 cells with DADS, one of the major organosulfur molecules present in our extract [38] and known to downregulate invasive potential of breast tumor cells with ER+ or triple-negative phenotypes [41,42]. As no data are present in the literature concerning the use of DADS on HER2+ cells, we treated cells for 72 h with various concentrations of the garlic compounds, including the one considered the most efficient for other breast tumor cells phenotypes. Apart from the lowest DADS concentration, a dose-dependent reduction in cell growth was revealed (Figure 3a,b), together with a decrease in the number of cells in the G0/G1 phases and their accumulation in the G2/M phases of cell cycle (Figure 3c,d). Treatment with DADS also induced a significant increase in the number of PI/annexin positive cells, even more pronounced than that induced by the garlic extract (Figure 3d–f), revealing in HER2+ cells the effects of DADS on cell growth and apoptosis similar to those described in other breast tumor phenotypes [25,42].

**Figure 4:**
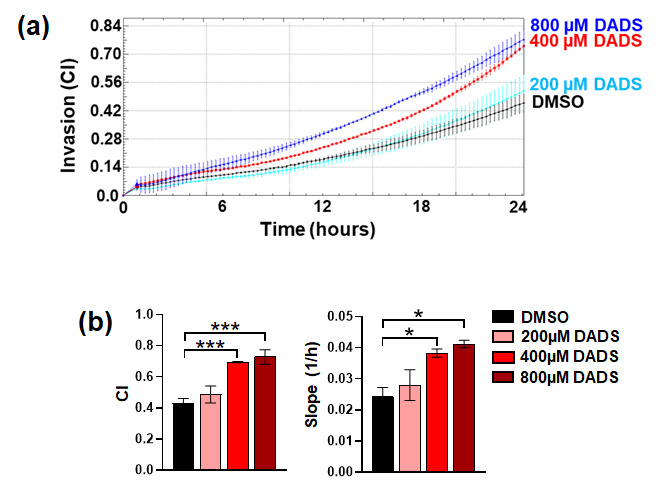
Effects of DADS on invasive capabilities of SKBR3 cells. After treatment for 72 h with DADS at the indicated concentrations, cells were subjected to evaluation of invasiveness (**a**). The analysis of cell index (CI) at 24 h of the assay, and of the slope, is also reported (**b**). Data represents the mean of the three separate experiments ± SD. * *p* < 0.05; *** *p* < 0.001 compared to the control (DMSO).

Based on the data obtained with LYO, we evaluated the effects of DADS on invasiveness of SKBR3 cells. We revealed that, as the garlic extract, DADS, induced a significant increase in invasive potential of the cell line (Figure 4a,b), even more pronounced than LYO at the higher evaluated concentrations, confirming that garlic derivatives have opposite effects on invasive potential of HER2+ with respect to the other breast tumor phenotypes.

### 3.3. The Effects of DADS on Invasive Potential of SKBR3 Cells Depend on the Duration of Treatment

**Figure 5:**
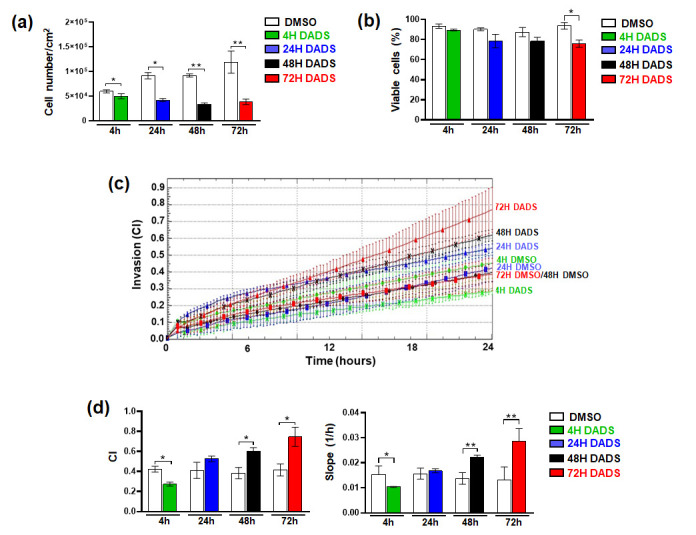
Effects of treatment duration with DADS of SKBR3 cells. Cells cultured in control conditions (DMSO) or treated with 400 µM DADS for the indicated times were subjected to evaluation of cell growth (**a**,**b**) and invasive capabilities (**c**). The analysis of cell index (CI) at 24 h of the assay, and of the slope is also reported (**d**). Data represents the mean of the three experiments ± SD. * *p* < 0.05; ** *p* < 0.01 compared to the control.

To assess if the increased invasive capability induced by DADS in SKBR3 cells is related to the duration of treatment, we exposed cells to the garlic derivative for a time between 4 and 72 h before evaluating their growth and invasive potential. We selected 400 µM as the DADS concentration to induce the highest effects on cell growth and invasiveness with the lowest cytotoxic effects, according to data from the literature on the use of this garlic derivative in other breast tumor cells [42]. As shown in Figure 5a,b, DADS substantially reduced cell growth at all the examined times, confirming its role in also blocking cell growth in breast tumor cells with an HER2+ phenotype. Interestingly, it exerted a peculiar time-dependent effect on invasive potential of SKBR3 cells. In particular, while 4 h of DADS was able to induce a significant reduction in invasiveness, no significant effects were revealed after 24 h, and longer treatments induced a substantial increase in invasive potential, which reached the maximum after 72 h (Figure 5c,d).

**Figure 6:**
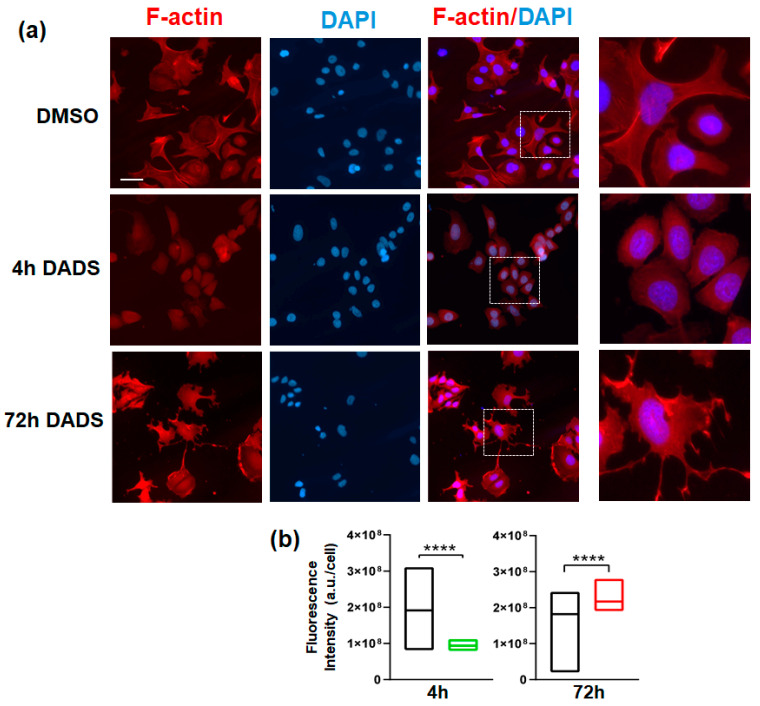
Effects of DADS on actin polymerization in SKBR3 cells. Representative fluorescence microscopy images of SKBR3 cells, growing on glass dishes for 4 or 72 h in control conditions (DMSO) or in the presence of DADS, and subjected to immunocytochemical analysis of F-actin after staining with TRITC-conjugated phalloidin (**a**). Nuclei were counterstained with DAPI. Merged images show the overlap of F-actin (red) and nuclei (blue) staining. On the right, zoomed white box-delimited images are shown. Bar: 20 µm. In (**b**), floating bars show the mean cell fluorescence intensity (a.u./cell). Black boxes: control condition. Green box: DADS treatment for 4 h. Red box: DADS treatment for 72 h. The data represents the mean of the four separate experiments ± SD. **** *p* < 0.0001 compared to the control.

To invade and metastasize, breast cancer cells must breach the basement membrane and then penetrate the dense extracellular environment of the mammary tissue [43]. To assess whether the peculiar effects of DADS on invasive capability of SKBR3 cells are accompanied by a substantial modification of cell morphology, we investigated actin polymerization, a crucial event in cancer cells, as the plasticity of the actin cytoskeleton is fundamental for cell migration, extracellular matrix invasion, and metastatic dissemination [44,45]. As reported in Figure 6a, in control cells, immunofluorescence for F-actin shows organization into well-defined stress fibers, structures that are crucial for maintaining cell shape and adherents’ junctions [46]. A brief treatment (4 h) with DADS induced a marked reorganization of the actin cytoskeleton, with loss of fibers in favor of a more diffuse and punctuated actin pattern (Figure 6a), indicative of depolymerization or dynamic redistribution of actin filaments. Conversely, after 72 h of DADS administration, the formation of new and more robust F-actin structures was revealed (Figure 6a), such as enhanced lamellipodia and filopodia, which are necessary to support the mesenchymal phenotype and increased cell motility [44,45,47]. Quantitative analysis of F-actin fluorescence intensity (Figure 6b) substantiated these morphological observations. A significant decrease in F-actin intensity was found after 4 h of treatment, consistent with the cytoskeletal disorganization that characterizes decreased motility, while a notable increase in total F-actin intensity per cell was recorded after 72 h, consistent with the morphological changes that describe increased invasive potential. Overall, the profound alterations observed in F-actin cytoskeleton organization, coupled with the modulation of its quantity, are highly consistent with the modification of invasive properties induced by DADS in this breast tumor cell model.

### 3.4. DADS Modulates Akt and β-Catenin in Parallel with SKBR3 Invasion

**Figure 7:**
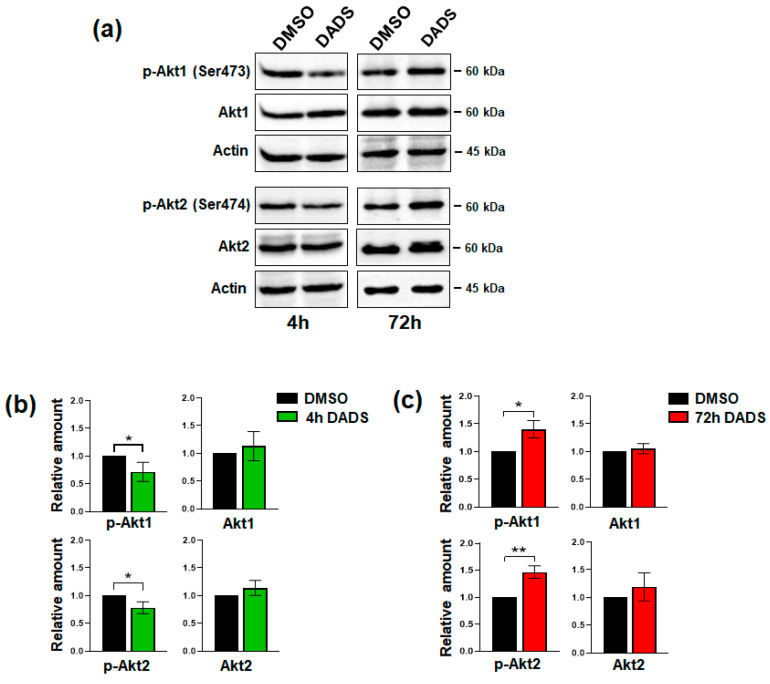
Effect of DADS on Akt status in SKBR3 cells. (**a**) Representative immunoblot analysis with the indicated antibodies of total lysates from SKBR3 cells after 4 and 72 h of culture in control conditions (DMSO) or in the presence of 400 µM DADS. Histograms, as deduced from the densitometry of Western blot bands normalized to actin, are shown (**b**,**c**). Data represents the mean of the three independent experiments ± SD. * *p* < 0.05; ** *p* < 0.01 compared to the control conditions taken as 1. The uncropped blots are shown in Supplementary File S1.

It was demonstrated that cells overexpressing HER2 exhibit increased Akt activity and that the PI3K/Akt signaling is a crucial modulator of the survival of HER2+ breast cancer cells [48,49]. Since it is known that Akt activation is related to tumor invasiveness in breast cancer, in which the activated protein has been correlated with F-actin organization and levels [49,50], we investigated whether the time-dependent changes in invasiveness induced by DADS in our HER2+ cell model were associated with a modulation of Akt status. Specifically, we analyzed Akt1 and Akt2, the two isoforms most closely related to invasiveness in breast tumors [51,52] and that we found modified by garlic extract in other breast tumor cells [33]. Using immunochemical analysis, we established that after 4 h of DADS administration, corresponding to a decrease in invasiveness, the phosphorylation of both Akt isoforms was downregulated (Figure 7). Conversely, after 72 h of treatment, where an increase in invasiveness was observed, the phosphorylation of both Akt isozymes were upregulated. The total Akt1 and Akt2 levels remained unchanged in all experimental conditions (Figure 7).

**Figure 8:**
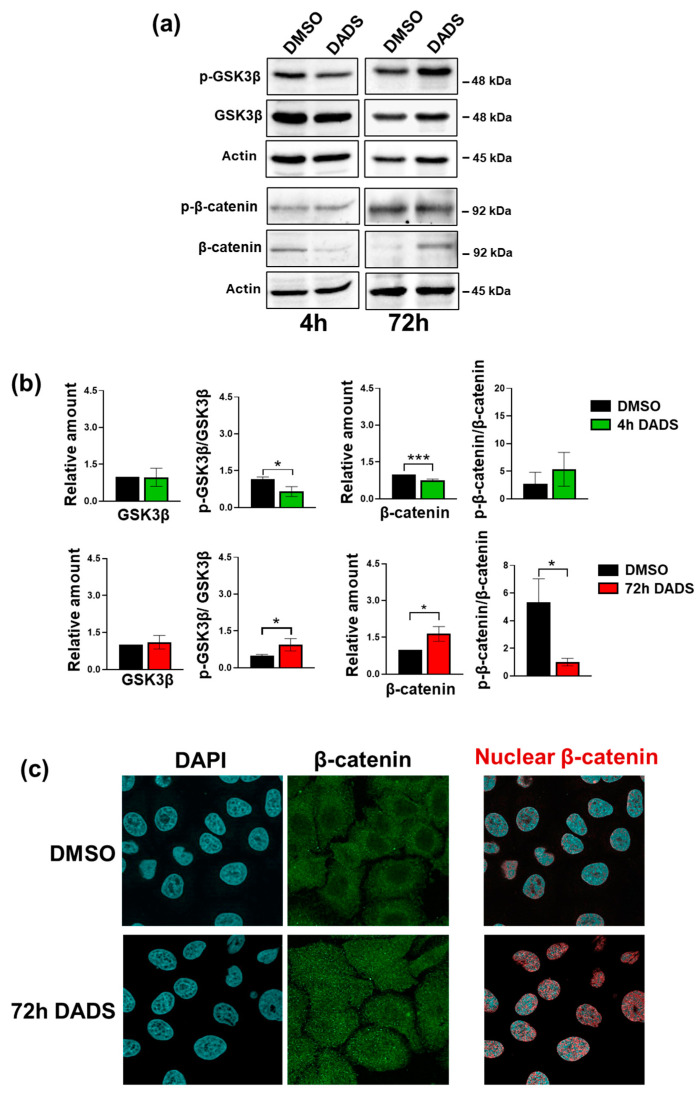
Effect of DADS on p-GSK3β/β-catenin status in SKBR3 cells. (**a**) Representative immunoblot analysis with the indicated antibodies of total lysates from SKBR3 cells after 4 and 72 h of culture in control conditions (DMSO) or in the presence of 400 µM DADS. Histograms, as deduced from the densitometry of Western blot bands normalized to actin are shown in (**b**). Data represents the mean of the three experiments ± SD. * *p* < 0.05; *** *p* < 0.001 compared to the control cells taken as 1. In (**c**), representative confocal images of SKBR3 cells treated with DADS for 72 h and stained with the antibody against β-catenin (green). DAPI was used to counterstain the nucleus (blue). On the right, the overlap of nuclear β-catenin (red points) with nuclei (blue) is shown. Magnification = 63×. The uncropped blots are shown in Supplementary File S1.

Assuming that the morphological changes induced by DADS in SKBR3 are associated with mesenchymal-related changes and based on the knowledge that the Akt pathways in breast tumor cells include the GSK3β/β-catenin axis [33,53], we explored the effects of the garlic derivative on β-catenin, whose overexpression has been shown to increase the activation of HER2 and HER3 receptors, leading to larger tumor sizes [19]. Using immunochemical analysis, we revealed that treatment for 4 h with DADS is sufficient to downregulate the β-catenin amount, while 72 h administration induced an increase in the protein levels (Figure 8a,c). The use of an anti-phospho-β-catenin antibody allowed us to assess the impact of DADS in that time-frame (Figure 8a,c). While the amount of GSK3β was unaffected by DADS administration, its phosphorylation level significantly decreased after 4 h and increased after 72 h of DADS treatment (Figure 8b,c), paralleling the effects of the garlic derivative on Akt.

Since it is known that the function of β-catenin varies depending on its location inside the cell [54], we performed a confocal immunofluorescence analysis of β-catenin in SKBR3 cells after a 72 h treatment with DADS. As shown in Figure 8c, under control conditions, β-catenin is mainly in the cytoplasm, with only a slight amount in the nuclear compartment. Conversely, after 72 h of treatment with DADS, β-catenin showed a strong accumulation inside the nucleus (Figure 8c), where it is known to be acting in the transcription of genes involved in tumor malignancy [55], providing a potential explanation for the increased invasiveness induced by DADS in our cell model.

**Figure 9:**
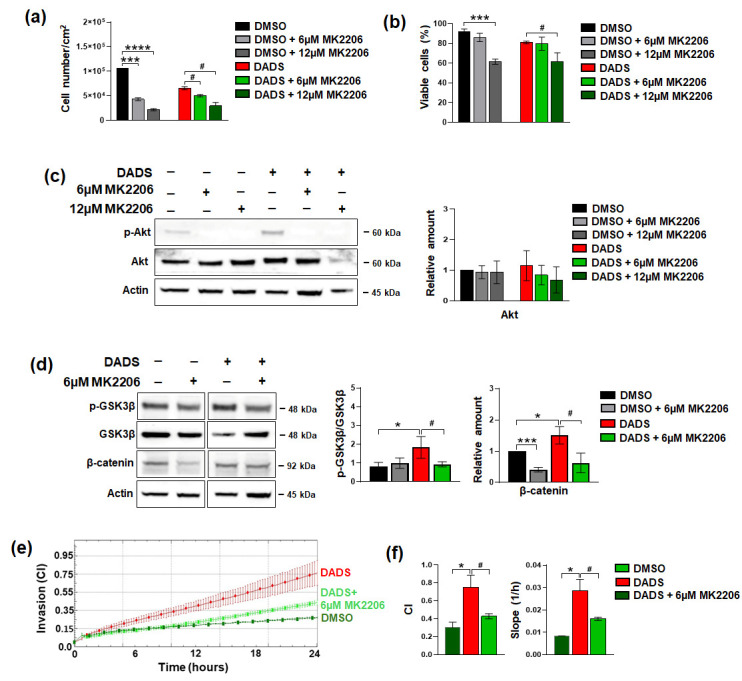
Effects of Akt inhibition on DADS impacts on SKBR3 cells. SKBR3 Cells were cultured in control conditions (DMSO) or treated with 400 µM DADS for 72 h in the presence of MK2206 at the indicated concentrations and subjected to evaluation of cell growth (**a**,**b**), Western blot analysis with the indicated antibodies (**c**,**d**), and invasive capabilities (**e**). The analysis of cell index (CI) at 24 h of the assay, and of the slope is also reported (**f**). Data represents the mean of the three experiments ± SD. * *p* < 0.05; *** *p* < 0.001; **** *p* < 0.0001 compared to the control. # *p* < 0.05 compare to DADS tratement. The uncropped blots are shown in Supplementary File S1.

The above reported results suggest the existence of an Akt/GSK3β/β-catenin axis by means of which DADS modulates invasive potential of SKBR3 cells. To ascertain that the Akt signaling is at the basis of the effects of the garlic derivative in our HER2+ overexpressing cell model, we used MK2206, an allosteric Akt inhibitor preventing its activation, during treatment with DADS for 72 h. As shown in Figure 9, the two MK2206 concentrations we tested, based on data from the literature [33], exerted a dose-dependent reduction in cell growth, both in control conditions and in DADS treated cells (Figure 9a,b), in parallel with the almost complete inhibition of Akt phosphorylation (Figure 9c).

As shown in Figure 9d, the Akt inhibitor completely counteracted the increase in pGSK3 and β-catenin induced by DADS, as well as the induced invasive potential (Figure 9e,f), indicating the complete dependence on Akt activation of the effects of this garlic derivative on invasive potential of our HER2+ cell model.

### 3.5. Impacts on Patients’ Survival of β-Catenin Levels in Their HER2+ Tumors

**Figure 10:**
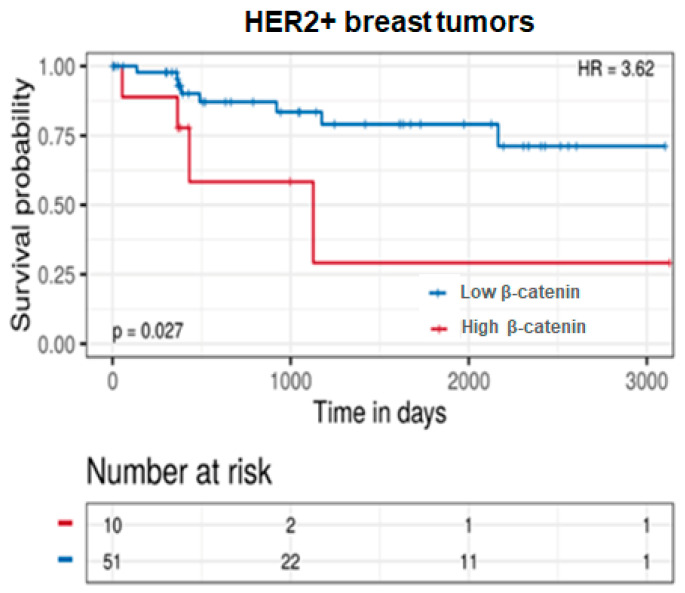
Impact of β-catenin levels on the follow-up of patients with HER2+ tumors. Progression-free survival (PFS) of patients with breast tumors overexpressing HER2, in relation to β-catenin expression in primary neoplasia. Survival curves were generated by separating the protein levels into two groups based on the lowest *p*-value.

Currently, there are few studies correlating the levels of β-catenin with the survival of patients with breast cancer and, instead, mainly take into consideration the specific membrane or nuclear localization of the protein [54,56]. Based on the above reported in vitro experiments, correlating total β-catenin in HER2+ tumor cells with their invasive potential, we explored the relationship between patient’s follow-up and total β-catenin in their breast tumors. We used TRGAted, a web tool for survival analysis [35] based on protein data from The Cancer Genome Atlas (TCGA) to examine the progression-free survival (PFS) of patients with HER2+ tumors in relation to β-catenin expression. The analysis included 61 patients with HER2+ tumors at all stages, stratified by β-catenin protein levels. As shown in Figure 10, patients with high levels of β-catenin in their tumors have a significantly worse PFS than patients with HER2 tumors showing low levels of β-catenin. Despite the small cohort and the failure to evaluate confounding factors, this analysis suggests that total β-catenin expression in primary tumors could be directly associated with the risk of disease progression in this breast tumor phenotype.

## 4. Discussion

Preclinical studies suggest that specific nutrients could slow tumor progression and enhance the effectiveness of anti-cancer therapies, offering, through diet, new possibilities to positively influence patient outcomes [57,58,59]. In this context, natural compounds are regarded with ever-increasing interest as a potential adjuvant to conventional drugs in designing oncological treatment strategies [21,22,23].

Several in vivo studies have demonstrated that organosulfur compounds derived from garlic such as ajoene, diallyl sulfide (DAS), DADS, and diallyl trisulfide (DATS) could offer promising experimental therapeutic options for melanoma, leukemia, prostate cancer, lung cancer, and hepatocellular carcinoma [30,60]. Concerning breast tumors, several data indicate that garlic is effective in protecting against their insurgence, in counteracting their aggressiveness, and in preventing the malignant progression of non-invasive breast tumor-derived cells. Using both in vitro and in vivo animal models it has been widely demonstrated that garlic derivatives induce apoptosis and reduce the invasive potential of ER+ and TNBC cells [25,30,31,32,41,61], but the current literature lacks investigation into their effect on HER2+ breast cancers. The aim of this study was therefore to explore the potential role of garlic-derived compounds on breast tumor cells with this specific phenotype. We first treated the MDA-MB-453 and SKBR3 cell lines, which were ER/PR-negative and expressing diverse levels of HER2 [62], with a lyophilized hydroalcoholic garlic extract containing a mixture of organosulfur compounds such as DAS, DADS, DATS, and resulted efficient in reducing cell growth and invasive potential of breast tumor cells with ER+ and TNBC phenotypes [31,32,38]. We assessed that, as in the other breast tumor phenotypes, our extract reduces cells growth and induces apoptosis in HER2+ breast tumor cells, while, unexpectedly, we revealed its ability to promote the invasive potential of both cell lines, although more accentuated in the SKBR3 cells, expressing the highest HER2 level.

Given the wide range of bioactive molecules in our garlic extract [38], and excluding the notion that that the observed effects on the ability of cells to pass through Matrigel were due to any contamination, we investigated whether specific garlic derivatives might be responsible for the increased invasiveness of our cell model. We focused on DADS, one of the most bioactive garlic organosulfur compounds [30,63], and evaluated its effects in the range of concentrations suggested by the literature for other cellular models of breast tumor. We revealed that, in parallel with the expected concentration-dependent reduction in cell growth and induction of apoptosis, DADS plays a peculiar effect on invasive potential of SKBR3 cells. In fact, invasive potential of our cell line was unaffected by the lower examined DADS concentration, but, apart from a slight but significant decrease in invasive potential after short exposure (4 h), starting from 48 h of treatment, cells showed a time and concentration dependent increase in invasiveness. These data, on one hand assessed that HER2+ breast tumor cells are less sensitive to DADS than other breast tumor phenotypes, [41,42] and other tumor cell models [64,65,66], on the other hand, revealed that their invasive potential increases when treated with DADS at concentrations useful for reducing invasiveness in other tumor models [41,42,67], underscoring, for the first time, a potential adverse effect of the use of garlic compounds on breast cancer cells

The observed morphological modifications and actin cytoskeleton reorganization confirmed that the increased invasive potential induced by DADS is the result of their improved ability to form protrusions, essential for extracellular matrix (ECM) invasion [46]. Notably, invadopodium formation is often regulated by the oncogenic tyrosine kinase Src, whose activity can be stimulated by HER2 [45], suggesting that the effects of DADS on our cell model might involve this receptor. This hypothesis could justify the lesser effect of garlic derivatives on MDA-MB-453, expressing HER2 at lower levels than SKBR3 cells.

To understand the molecular basis of the observed phenomenon, we investigated the Akt signaling, a key regulator of cancer cell invasion by acting on the amount and organization of F-actin [50], and we found it to be downregulated by garlic extract [33]. Akt activation is crucial in the HER2 signaling [49] and is down-modulated by specific garlic derivatives in ER+ and TNBC breast tumor phenotypes and by DADS in various cancer cells [30]. Here we found that, at variance with its effects in other breast tumor phenotypes, acute DADS administration in HER2-overexpressing cells induced down-modulation, while longer treatments up-modulate the activation of both Akt1 and Akt2, known to promote growth and invasive potential through partially overlapping and/or opposite effects [50,52]. Remarkably, in HER2+, as in TNBC cells, we highlighted that Akt modulations induced by garlic derivatives directly correlate with the levels of β-catenin, a critical regulator of cancer malignancy [18,19,20], reinforcing the existence, also in this cell model, of a direct relationship between Akt/β-catenin and invasion. Notably, in HER2+ cells we assessed the existence of an Akt/GSK3β/β-catenin axis that can be up-modulated by DADS and that culminates in the accumulation of β-catenin inside the cell nucleus, where it is known to affect expression of genes involved in various cancer related events [68,69]. Since nuclear β-catenin is associated with enhanced invasiveness and metastatic potential of tumor cells, our data indicate that DADS may promote these processes in HER2+ breast tumor cells inducing an increase in, and nuclear accumulation of, this member of the Wnt signaling.

A relationship between β-catenin and DADS has been reported in various cancers and in TNBC breast tumor cells [41]. We demonstrated here that this protein is one of the main players in the effects of DADS in SKBR3 cells, although very few data clearly correlated the levels of β-catenin with the prognosis of patients with HER2+ breast cancer. Li et al. [56] examined the intracellular localization of β-catenin, showing that the invasive breast cancer displayed significantly increased cytoplasmic and nuclear staining of β-catenin with respect to normal tissues. They also revealed that cytoplasmic and/or nuclear localization of the protein is significantly associated with an adverse outcome in each breast tumor molecular subtype, including HER2+, confirming the role of nuclear β-catenin in malignancy of breast tumor cells. In our cell model, β-catenin is mainly localized in the cytoplasm, in line with the tumor phenotype, and the nuclear accumulation induced by DADS seems to be the result of its reduced degradation due to up-modulation of the Akt/GSK3β signaling, suggesting that the total amount of the protein could also have some significance. To try to translate our findings to a clinical context, we used the TRGAted web tool [35] to analyze protein data from the TCGA. Despite the low number of patients included in the analysis and the failure to evaluate confounding factors, we found that elevated levels of β-catenin in HER2+ primary tumors strongly correlate with low progression-free survival rate of patients. This, on the one hand confirms β-catenin as a potential negative prognostic factor in this specific breast tumor phenotype, and on the other hand indicates that a garlic derivative with well-demonstrated anti-tumor properties in various cancers can up-modulate a molecule negatively correlated to the tumor malignancy. Although the effects of DADS on β-catenin need to be verified in vivo, this event is of particular interest because in HER2-overexpressing cells the upregulation of β-catenin increases the activation of HER2 and HER3 [19], whose dimerization stimulates the activation of the PI3K/Akt pathway [49], which is, in turn, involved in tumor malignancy.

The peculiar effects of DADS that we observed in breast tumor cells overexpressing HER2, and the receptor-correlated signaling involved, suggest that this garlic compound may interfere with HER2 activity. This raises the question of whether the effect of DADS was related to the strength of treatment, in terms of doses and/or duration, as reported in the literature for some HER2 targeting substances, like Herceptin (Trastuzumab), for which several mechanisms of resistance, including the activation of Akt signaling, have been identified [70]. The selection in the SKBR3 population of the higher invasive cells by prolonged administration is another possibility, suggested by the reduced invasiveness induced by brief DADS treatments, which cannot affect cell growth and apoptosis.

Although the anti-cancer mechanism of DADS has been extensively well studied, its anti-tumor efficacy remains predominantly confined to in vitro animal models, with only fragmentary documentation of in vivo pharmacological performance. In fact, being a lipophilic compound, it is very difficult to assess its plasma concentration, metabolism, and the required dose in vivo, and, there are no published data on DADS (or other garlic derivatives) dosages, clinical safety, pharmacokinetics, and actual in vivo efficacy in breast cancer patients [30,70,71]. In addition, despite that up to now it has been reported that garlic has favorable effects against cancer in vitro and animal studies, a recent review of clinical trials [72] revealed an inverse association of garlic intake with stomach and colorectal cancer, and contradictory results on the relationship between the outcomes of various cancer and types of garlic intake. Our findings highlight the need for future studies aimed at evaluating the clinical use of DADS (and other natural compounds) to also consider that the same treatment (in terms of timing and concentration), in addition to having different effects on diverse cancers, could induce an opposite response in different phenotypes of the same tumor.

Although the results obtained are consistent, a possible limitation of our work could be the use of two cell lines with different genomic characteristics, which makes them not completely comparable. On the other hand, they were selected because they express different HER2 levels, representing two distinct scores of the receptor profile. In addition, the two cell lines show similar basal levels of Akt, whose activation status reflects their HER2 expression, which significantly impacts the response to HER2-targeted therapies and could explain the different intensity of their responses to garlic derivatives.

## 5. Conclusions

Although further investigations are needed to establish the mechanism responsible for the dual action of garlic derivatives in HER2+ tumor cells and to establish whether this phenomenon is reproducible in in vivo models, our results highlight an often-overlooked aspect that is the variability of the effects of natural compounds in complex diseases such as cancer. Particularly, breast tumors are characterized by high heterogeneity, various metabolic requirements, and response to microenvironments. In this context, the contemporary use of traditional therapy with dietary modification must be clinically evaluated.

## Data Availability

All data supporting the findings of this study are available on paper.

## Supplementary Materials

The following supporting information can be downloaded at: https://www.mdpi.com/article/10.3390/cancers17213572/s1, Figure S1: MDA-MB-453 and SKBR3 cell lines basal differences. (a) Reprensentative immunoblot analysis with the indicated antibodies of total lysatef from MDA-MB-453 and SKBR3 cells in control conditions. (b) Histograms as deduce from densitometry of WB bands normalized to actin. File S1: Western blot information.
