# Supplementary material for: Dual Role of Diallyl Disulfide (DADS) on Invasive Potential and β-Catenin Dynamics in HER2-Positive Breast Cancer Cells
Source: Cancers (Basel). 2025 Nov 5;17(21):3572. doi: 10.3390/cancers17213572 (PMC12608961; doi:10.3390/cancers17213572)

Original Images for Blots

For each experiment, treated sample and its respective control, used for comparative analysis and identified by a rectangle, were run on the same membrane and subjected to the same hybridization. Each independent biological experiment used for quantitative analysis is named “Repeat” and Repeat 1 correspond to the selected representative images reported in Figure 6A and 7A.

Membranes were cut for the simultaneous analysis of various proteins and to reduce the amounts of used antibodies.
Molecular weight markers (MW) are often evident in lanes present in the original images and are included in the raw data images. Unspecific bands are indicated with an arrow. Bands not relevant to the manuscript, related to treatments with different concentrations, different times or substances other than DADS could be present in the membranes analyzed. Unrelated bands, not included in the final Figures, have been marked with an “X”. In the figure the values of densitometrical analysis of Western blot bands are also reported as arbitrary units (a.u.).

Figure 7A: SKBR3 cells treated for 4h with DADS, original blot images

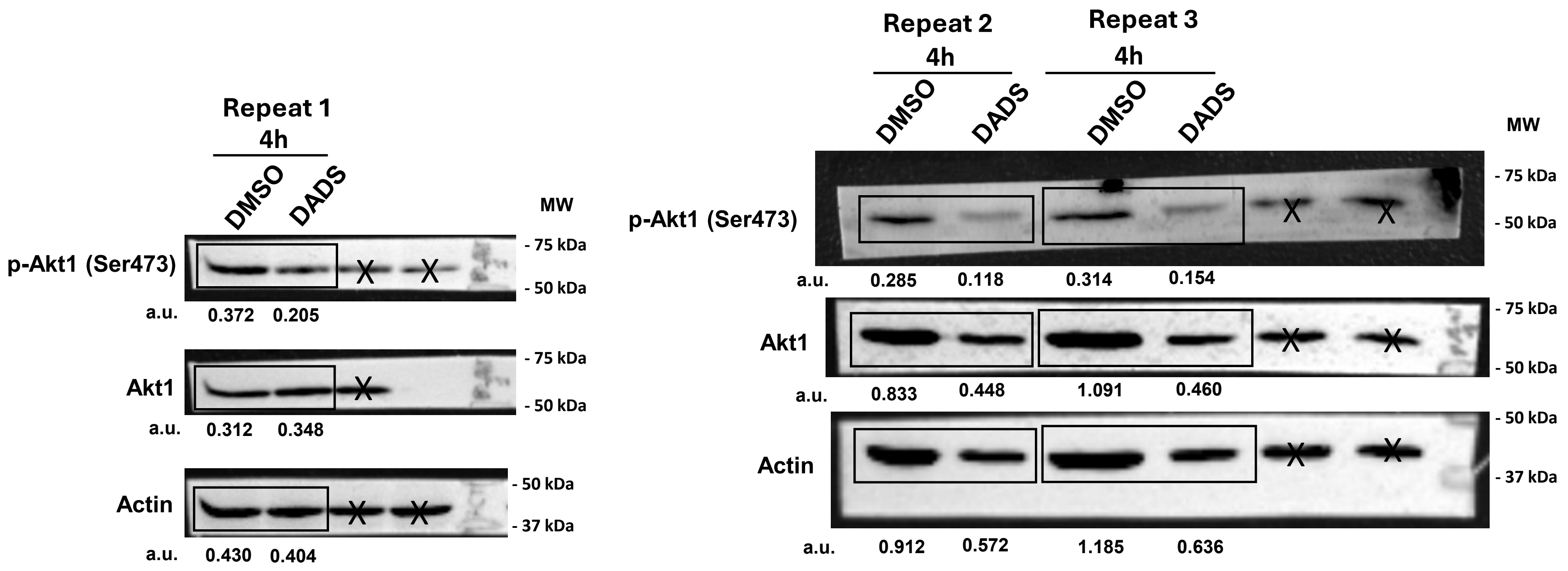

Figure 7A: SKBR3 cells treated for 72h with DADS, original blot images

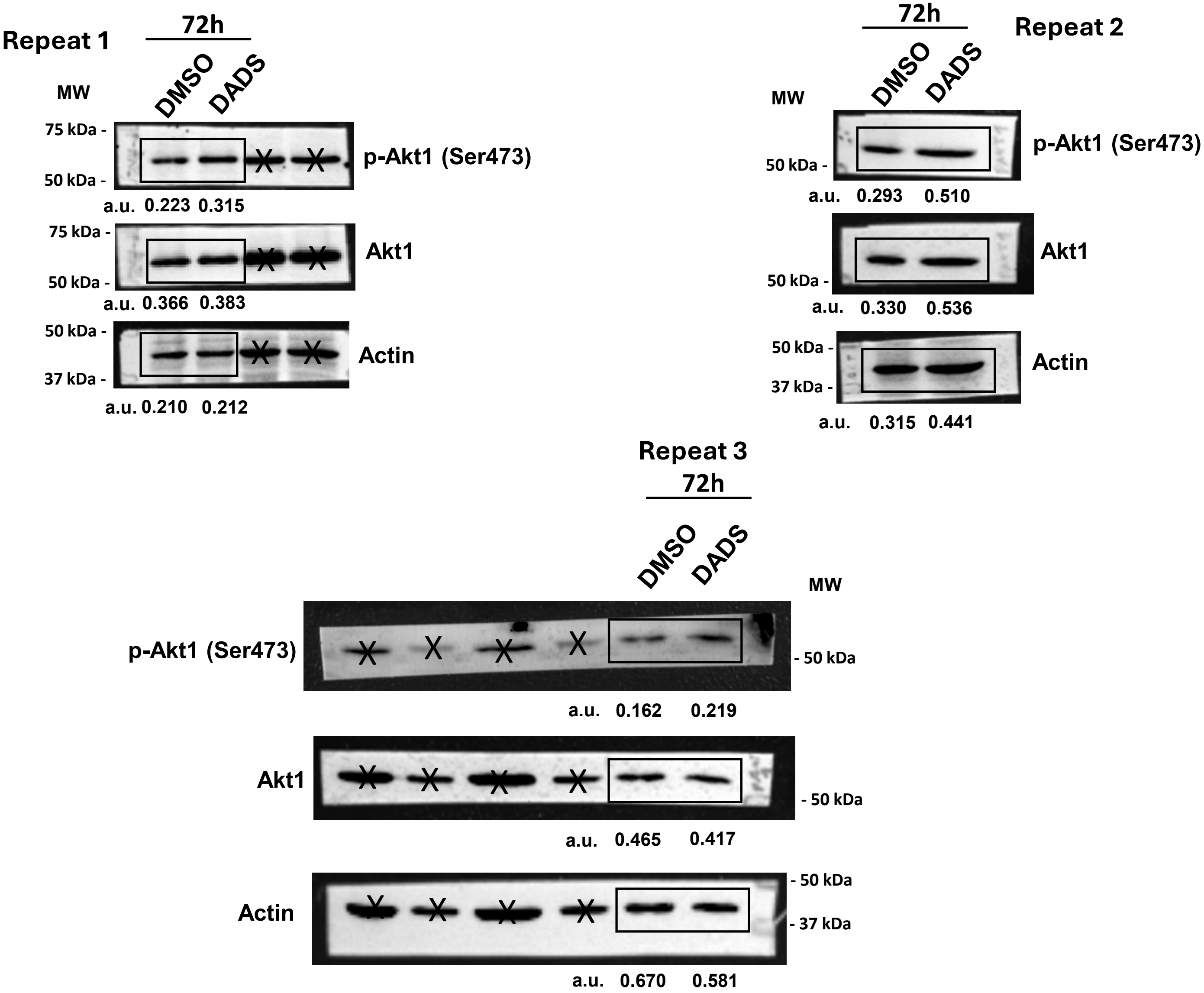

Figure 7A: SKBR3 cells treated for 4h with DADS, original blot images

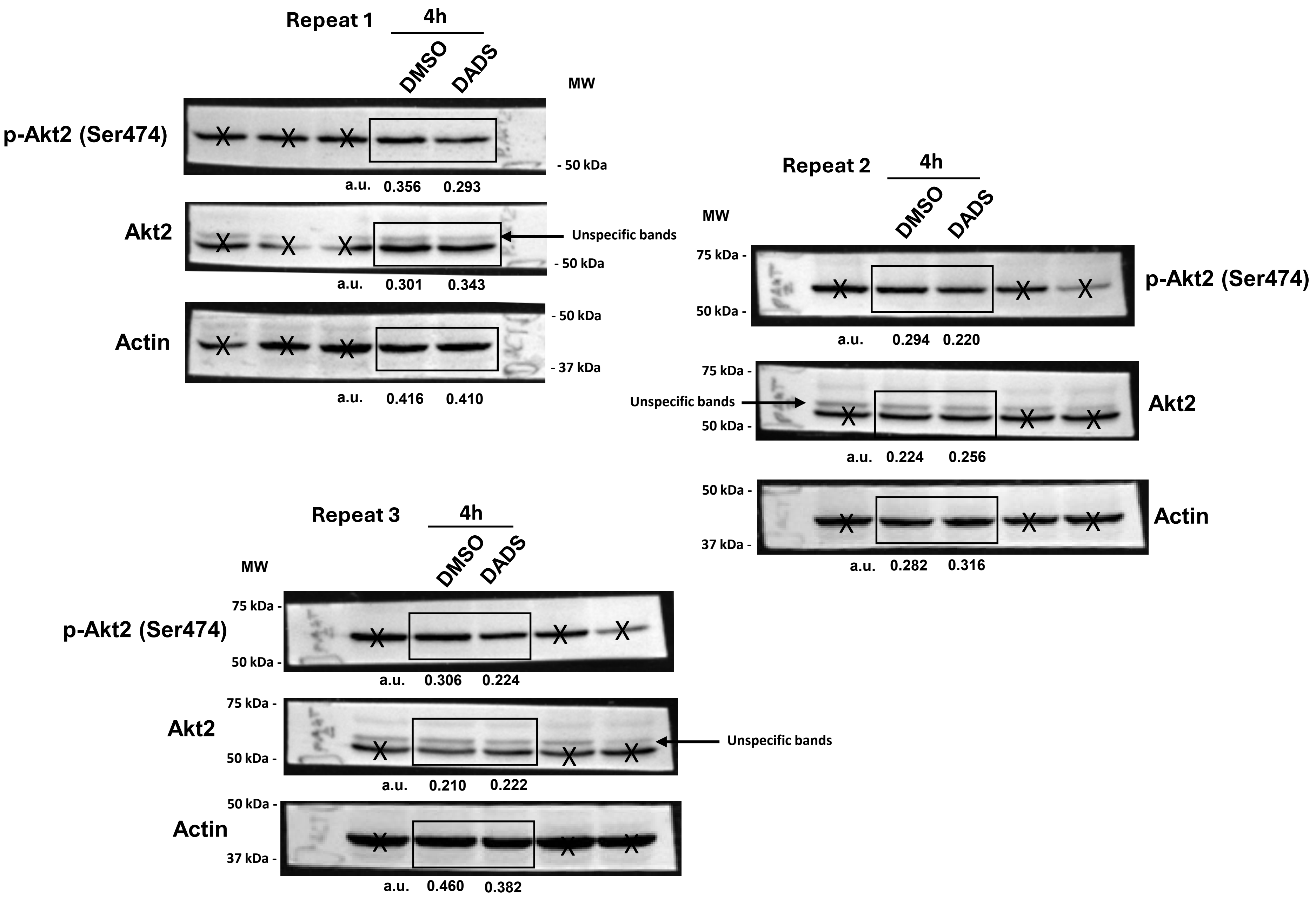

Figure 7A: SKBR3 cells treated for 72h with DADS, original blot images

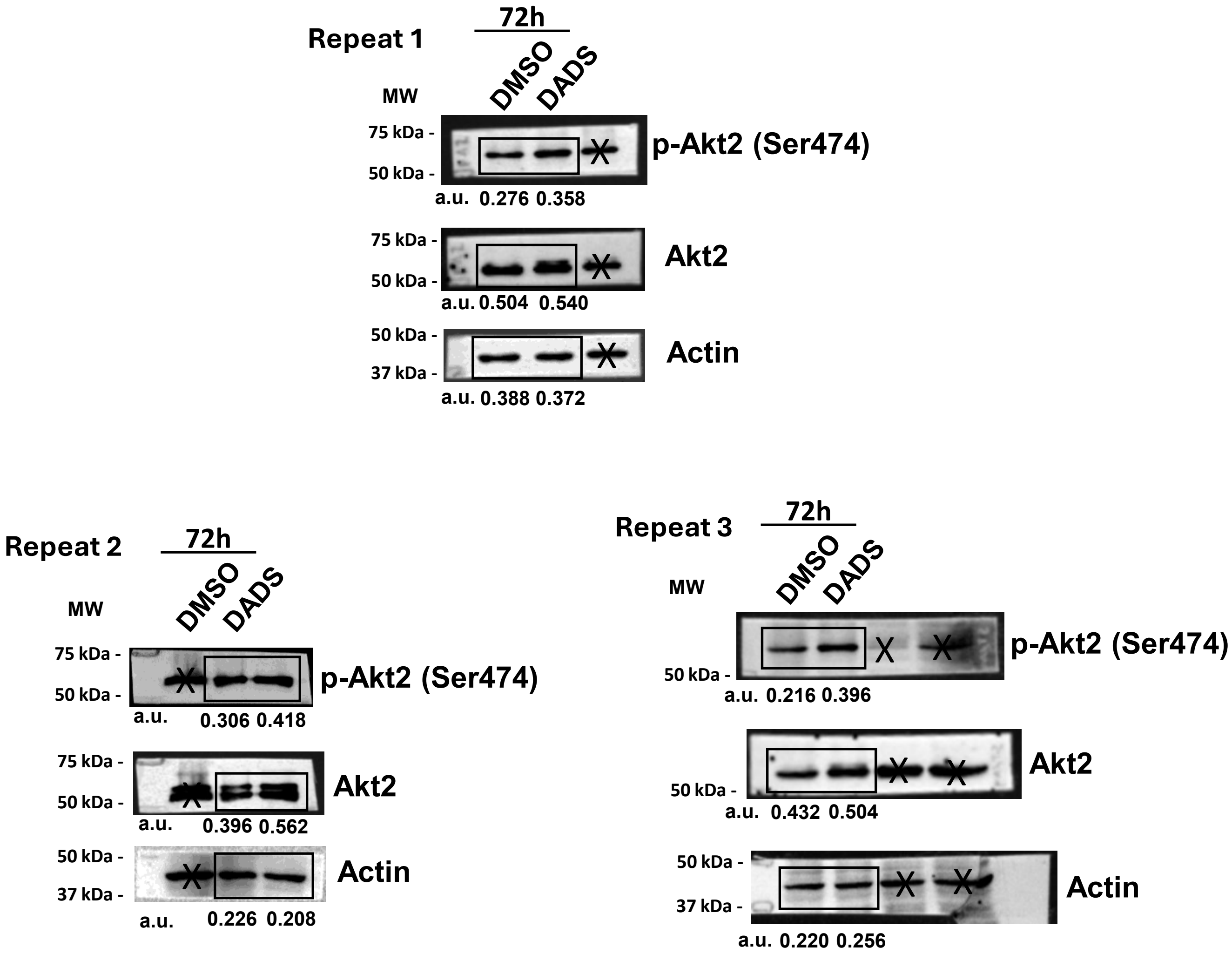

Figure 8A: SKBR3 cells treated for 4h with DADS, original blot images

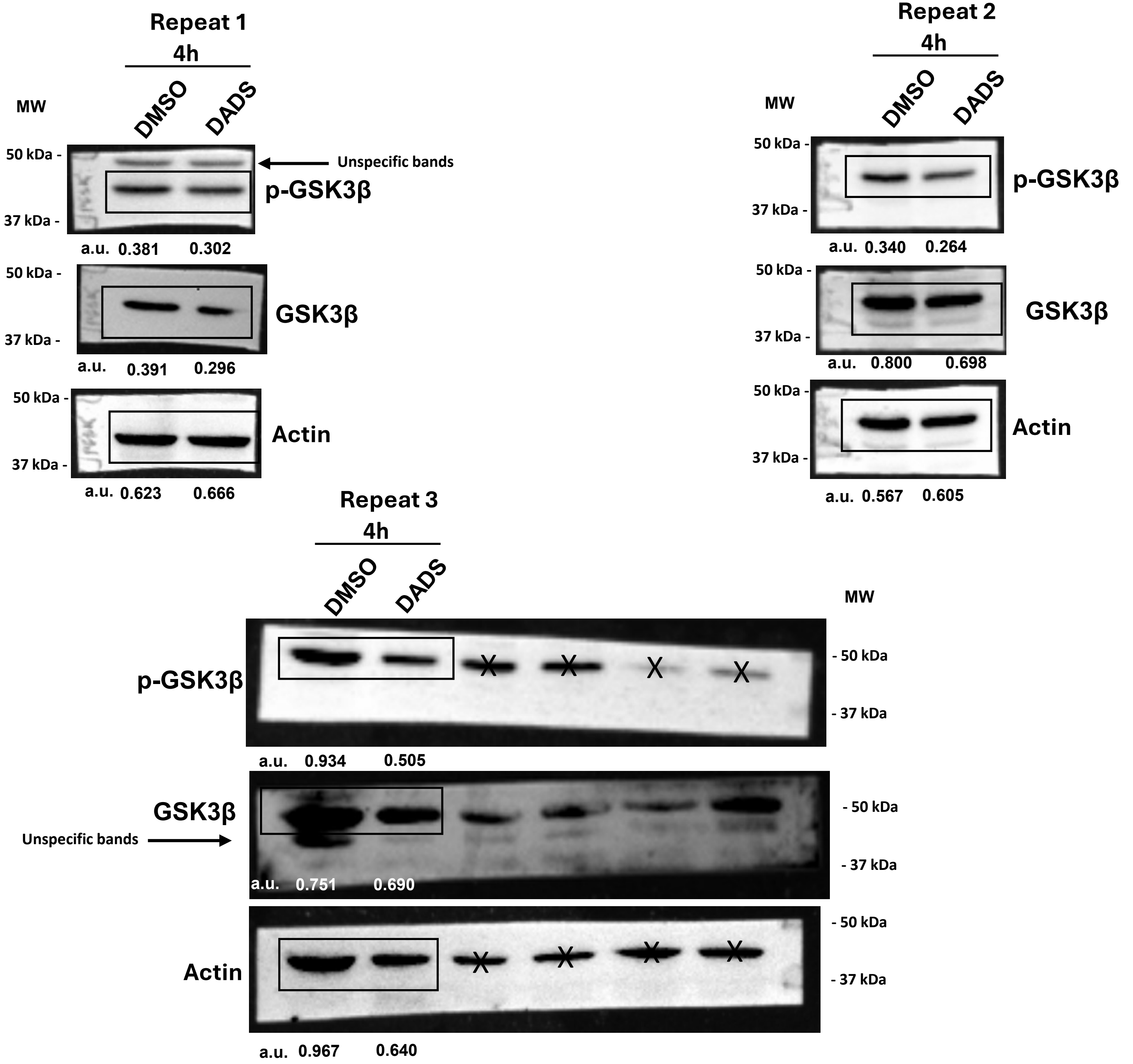

Figure 8A: SKBR3 cells treated for 72h with DADS, original blot images

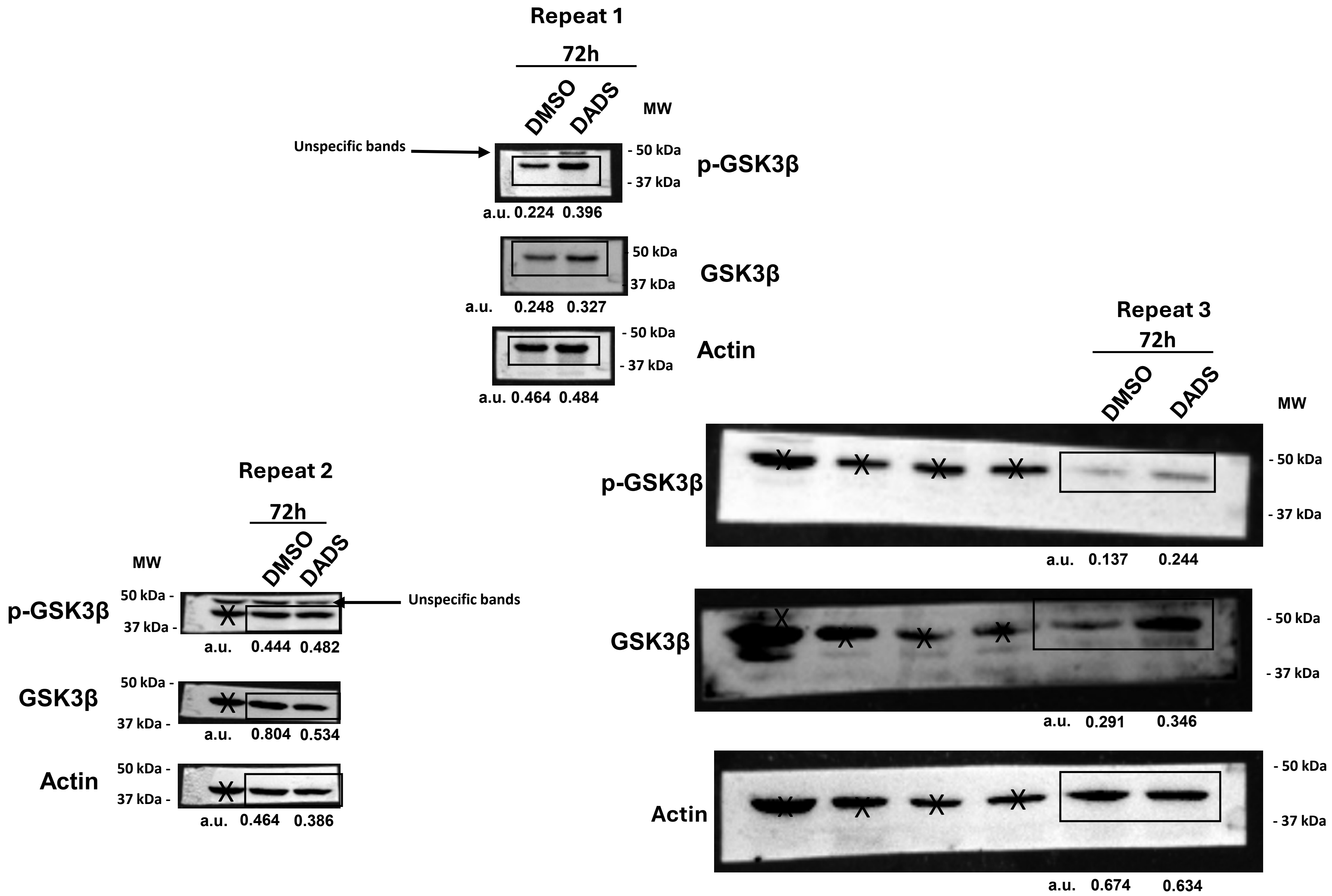

Figure 8A: SKBR3 cells treated for 4h with DADS, original blot images

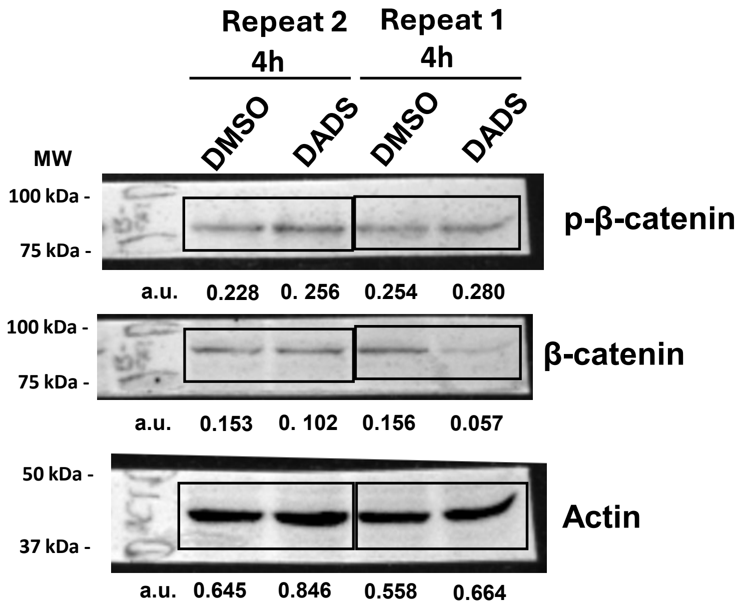

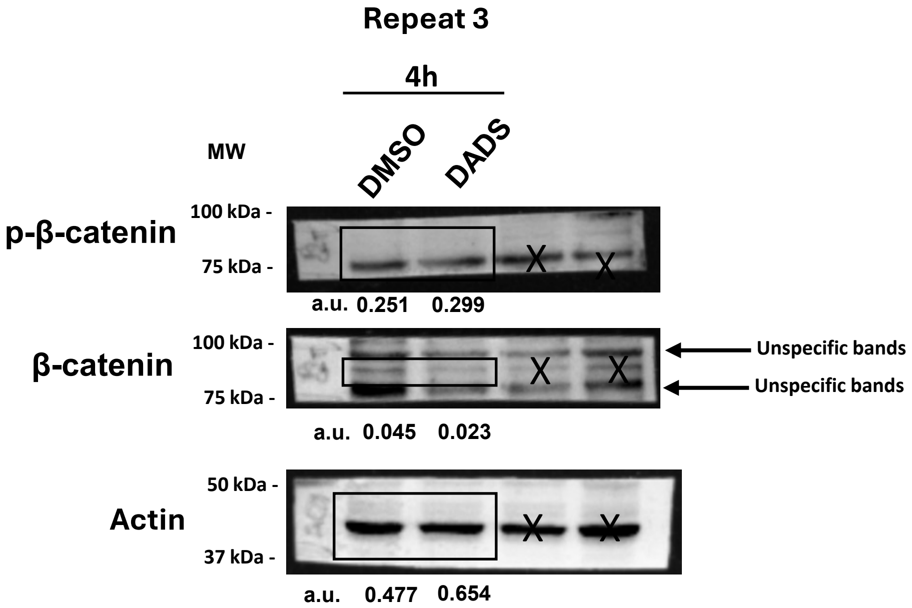

Figure 8A: SKBR3 cells treated for 72h with DADS, original blot images

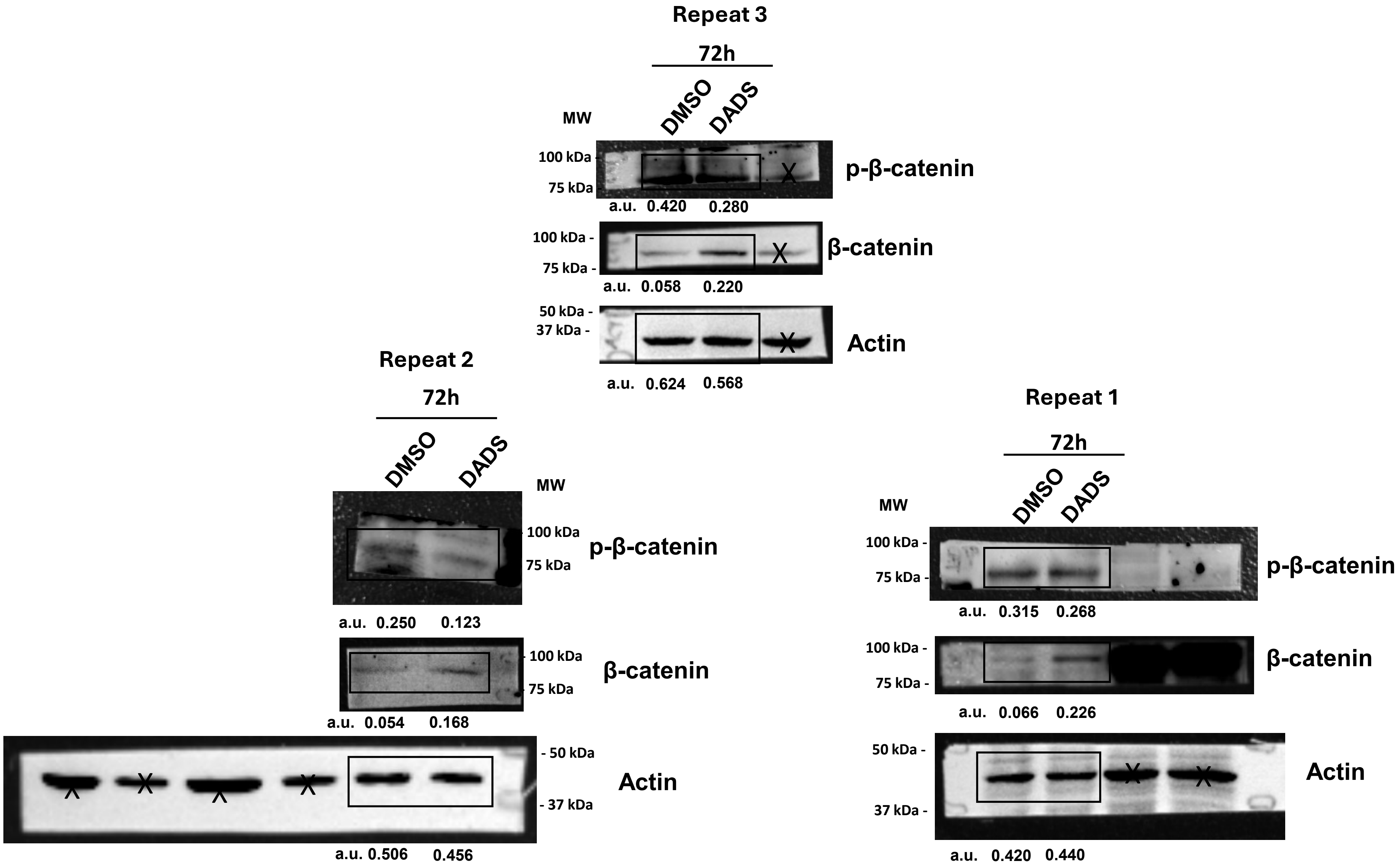

Figure 9C and 9D: SKBR3 cells treated or not for 72h with DADS and Akt inhibitor MK-2206, original blot images

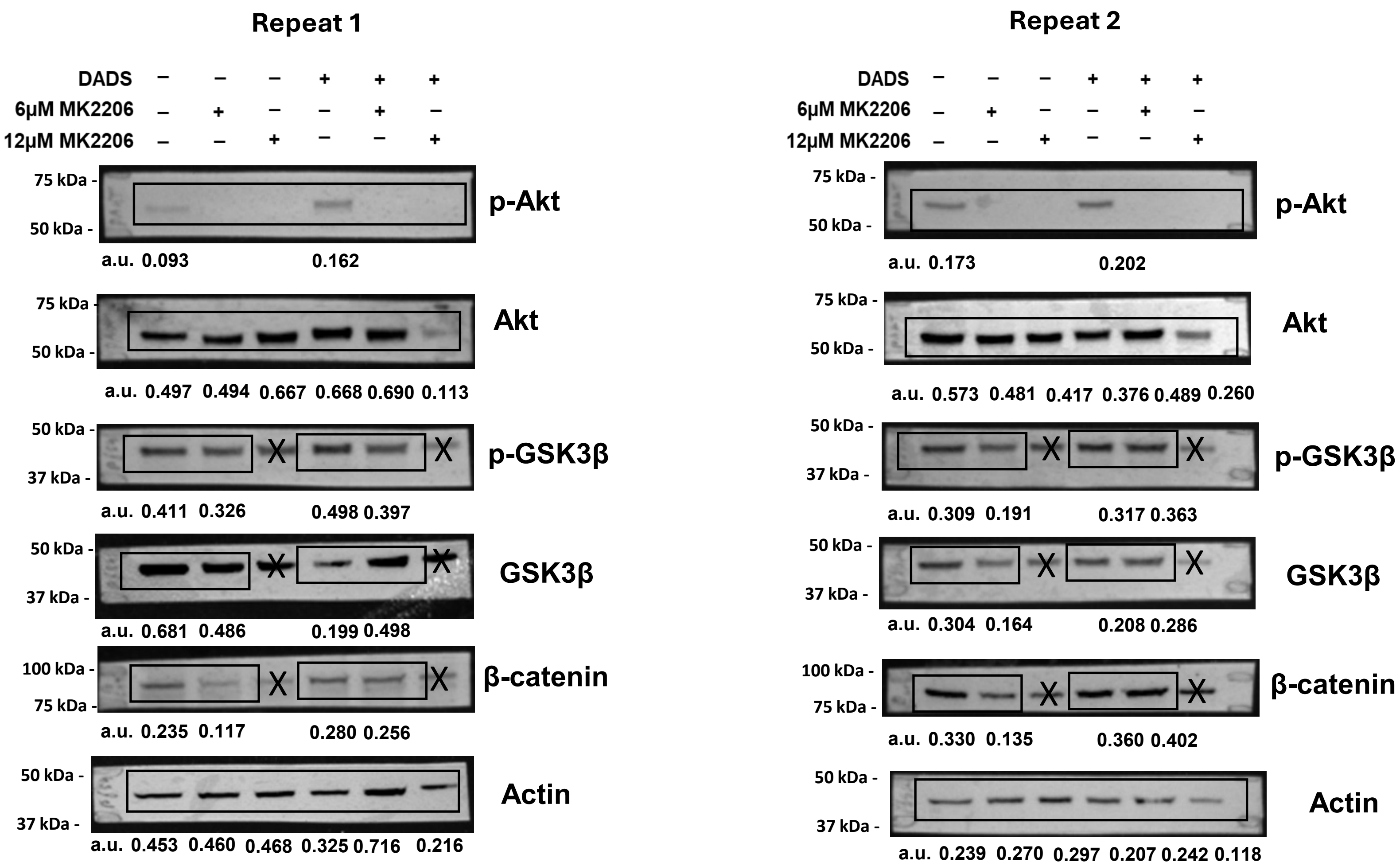

Figure 9C and 9D : SKBR3 cells treated or not for 72h with DADS and Akt inhibitor MK-2206, original blot images

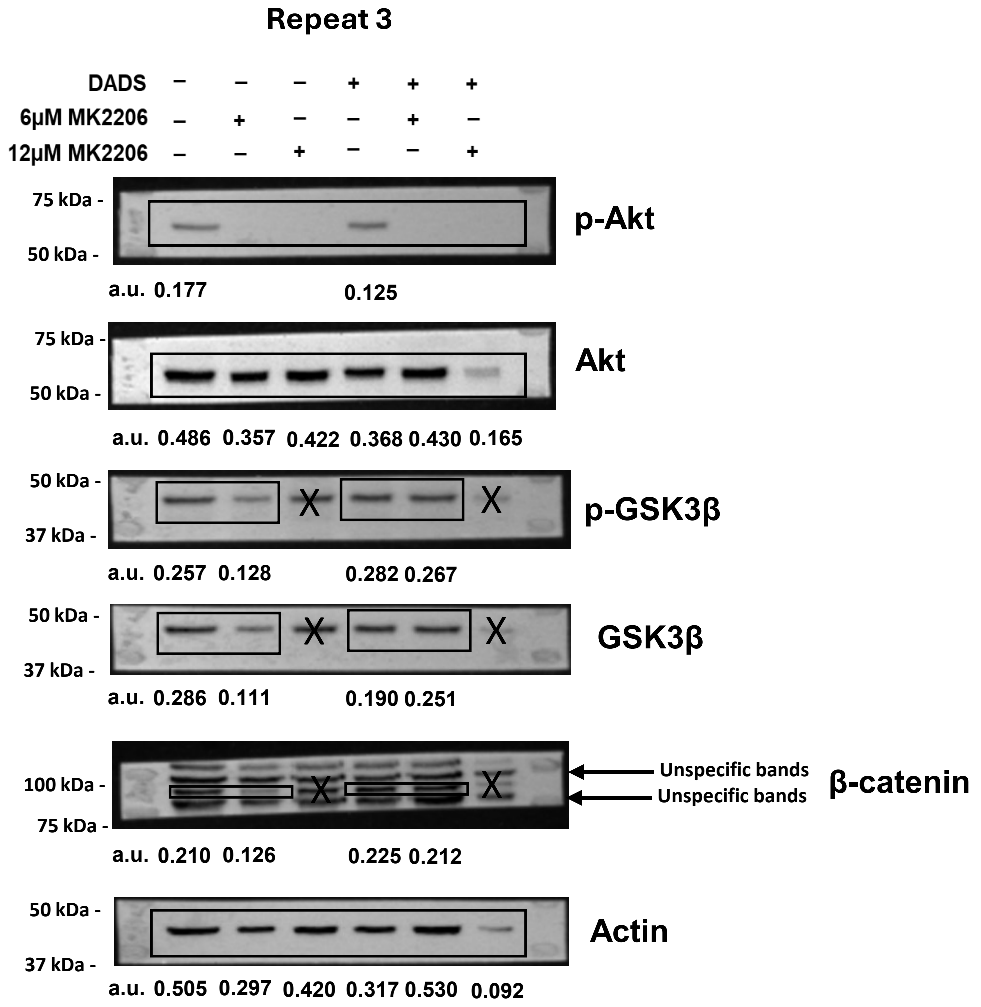

Supplementary Figure 1

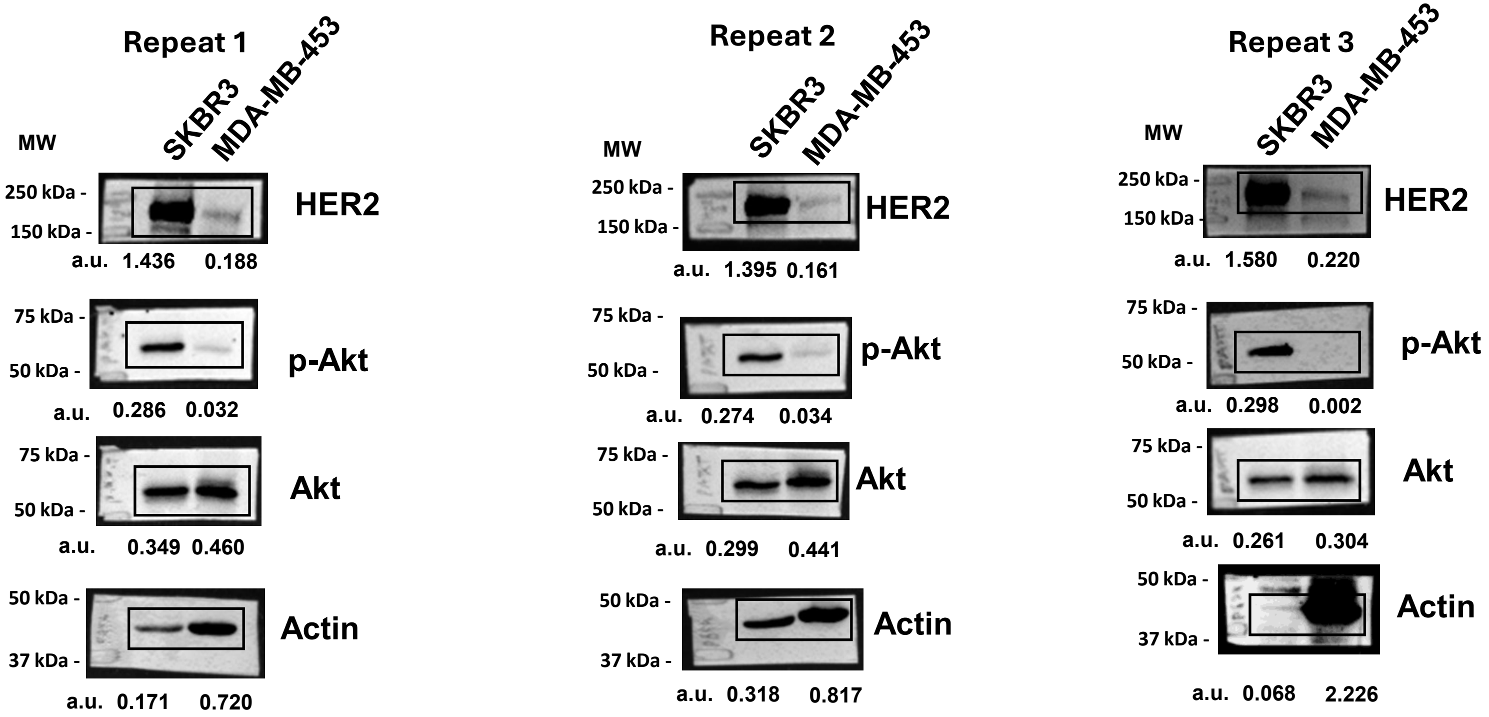

Supplement: Supplementary file 1 [file cancers-17-03572-s001.zip › cancers-3774201-supplementary File S1.pdf]
