# Supplementary material for: Dual Role of Diallyl Disulfide (DADS) on Invasive Potential and β-Catenin Dynamics in HER2-Positive Breast Cancer Cells
Source: Cancers (Basel). 2025 Nov 5;17(21):3572. doi: 10.3390/cancers17213572 (PMC12608961; doi:10.3390/cancers17213572)
Supplement: Supplementary file 1 [file cancers-17-03572-s001.zip › SUPPLEMENTARY FIGURE S1.pdf]

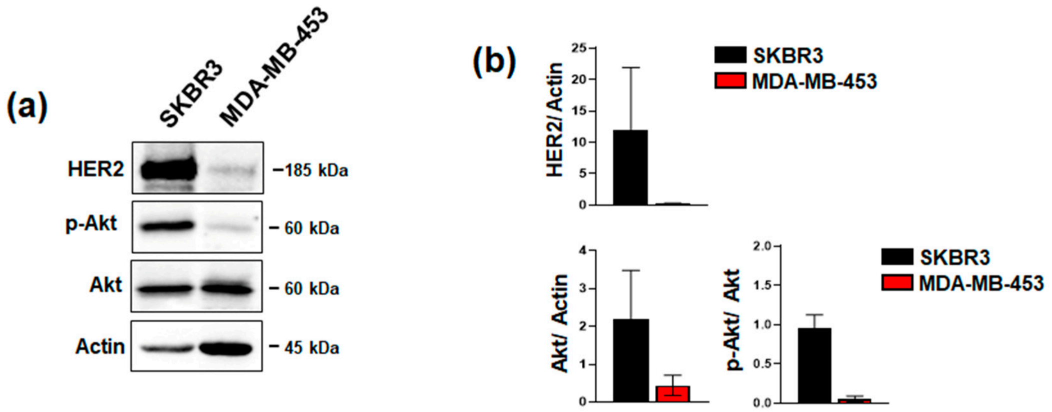

Figure S1. MDA-MB-453 and SKBR3 cell lines basal differences. (a) Reprensentative immunoblot analysis with the indicated antibodies of total lysatef from MDA-MB-453 and SKBR3 cells in control conditions. (b) Histograms as deduce from densitometry of WB bands normalized to actin.
